# Wide-Baseline Stereo-Based Obstacle Mapping for Unmanned Surface Vehicles

**DOI:** 10.3390/s18041085

**Published:** 2018-04-04

**Authors:** Xiaozheng Mou, Han Wang

**Affiliations:** School of Electrical and Electronic Engineering, Nanyang Technological University, Singapore 639798, Singapore; hw@ntu.edu.sg

**Keywords:** obstacle mapping, wide-baseline stereo, visual odometry, unmanned surface vehicle

## Abstract

This paper proposes a wide-baseline stereo-based static obstacle mapping approach for unmanned surface vehicles (USVs). The proposed approach eliminates the complicated calibration work and the bulky rig in our previous binocular stereo system, and raises the ranging ability from 500 to 1000 m with a even larger baseline obtained from the motion of USVs. Integrating a monocular camera with GPS and compass information in this proposed system, the world locations of the detected static obstacles are reconstructed while the USV is traveling, and an obstacle map is then built. To achieve more accurate and robust performance, multiple pairs of frames are leveraged to synthesize the final reconstruction results in a weighting model. Experimental results based on our own dataset demonstrate the high efficiency of our system. To the best of our knowledge, we are the first to address the task of wide-baseline stereo-based obstacle mapping in a maritime environment.

## 1. Introduction

In a binocular stereo vision system, the depth *Z* of a point is computed as

(1) $$Z=\frac{fB}{d}$$

where *B* is the baseline; *f* is the focal length of the camera in pixels; *d* is the disparity value. Assuming there is a small disparity error Δd added to the disparity *d*, then the depth error ΔZ is derived as

(2) $$\Delta Z=\frac{Z^{2}}{fB}\Delta d.$$

In Equation (2), one can see that the depth error ΔZ is inversely proportional to the length of baseline *B* by supposing the focal length *f* of the camera is fixed. In other words, larger baseline yields smaller depth error at a same depth value. Thus, as in Equation (1), under the same disparity *d*, a larger depth *Z* can be obtained with a larger baseline *B*.

**Figure 1:**
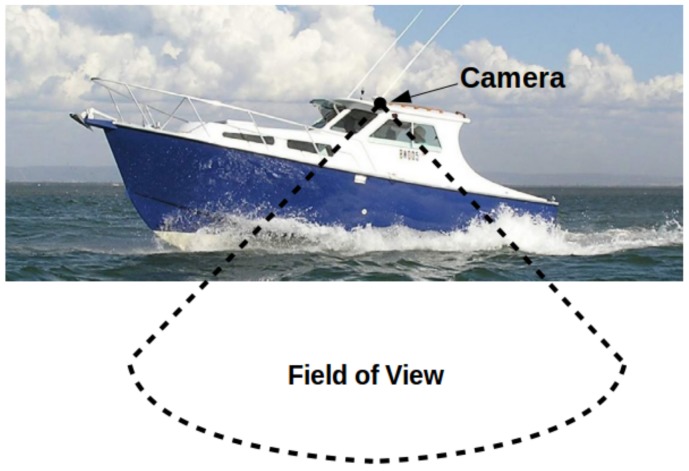
Proposed camera location on the boat.

In our previous work [1,2,3,4], we built a binocular stereo vision system with a very large baseline *B* and focal length *f*, and a ranging ability within 500 m was achieved. However, increasing the ranging ability of the binocular stereo vision system will result in an even bulkier structure on the boat and a problem for calibration. Moreover, binocular HD cameras require extensive amounts of data to be processed. To eliminate these problems, in this paper, we propose a wide-baseline stereo-based system, in which only one camera is used, and the depth is restored via the motion of the unmanned surface vehicle (USV). The motion of the USV can provide a large enough baseline, so that the ranging ability can reach as high as 1000+ m. Figure 1 illustrates the position of our monocular camera mounted on a USV. The camera is facing the side of the USV. In our proposed system, the USV is travelling at a high speed. The sensors include a single digital camera, a GPS device, and a compass.

To reconstruct and map the detected points from obstacles, it is necessary to know the visual odometry (VO) of the USV. Conventional SLAM methods [5,6] applied in unmanned ground vehicles (UGVs) and unmanned aerial vehicles (UAVs) for computing VO assume that many features can be detected in each frame; however, this is not suitable to the open sea environment, where, usually, very few features can be detected. Therefore, in this paper, we propose a new solution to compute the visual odometry of USVs in maritime environments. The translation of the camera is computed using recorded GPS data. The rotation of the camera is recovered by the detected horizon line (roll and pitch) and a compass or the IMU (yaw). After the VO is obtained, the depth of each feature point is calculated by matching the detected feature points between two frames. To achieve more accurate and robust performance, multiple frame pairs are leveraged to synthesize the final reconstruction results in a weighting model. Finally, these feature points from obstacles are projected to Google Maps.

The novelties in this paper mainly reside in computing the VO for USV via the detected horizon line, a magnetometer, and a GPS, rather than conventional VO methods. Furthermore, with the aid of computed VO, we simplify the problem of point reconstruction from 3D to 2D, which not only reduces the complexity of computation, but also improves the accuracy, because the height information from GPS is not always reliable. To the best of our knowledge, we are the first to address the task of wide-baseline stereo-based obstacle mapping in maritime environments.

The paper is structured as follows: Section 2 introduces the theory of motion parallax behind the proposed system. Section 3 describes our proposed approach for computing the VO of the USV. Section 4 reduces the point reconstruction problem from 3D to 2D. Section 5 briefly outlines the feature detection, tracking, and matching. Section 6 describes the proposed obstacle mapping with the synthesis of multiple frames. Experimental results on our own dataset are shown and discussed in Section 7. Section 8 draws conclusions.

## 2. Theory Behind the System

In a wide-baseline stereo-based system, a 3D motion of the camera causes an effective translation and rotation of the object relative to the camera, and the resulting image motion of points and lines reveal their 3D geometries. This fact is known as motion parallax, which is the main theory behind our proposed system.

**Figure 2:**
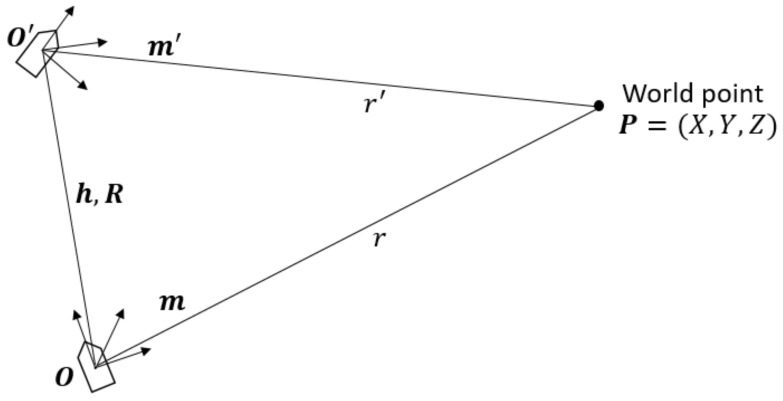
Illustration of motion parallax.

As shown in Figure 2, we suppose the camera mounted on a vehicle undergoes a translation h followed by a rotation R from *O* to $O^{\prime }$. A static world point *P* has been observed twice as (x,y) and $\left(x^{\prime },y^{\prime }\right)$, respectively, in the images. Therefore, we have the following vector relation:

(3) $$\vec{O^{\prime }P}=R^{-1}\left(\vec{OP}-\vec{OO^{\prime }}\right)$$

where $\vec{OP}$ and $\vec{OO^{\prime }}$ are determined at the local frame with the origin *O*, while $\vec{O^{\prime }P}$ is given in the local frame with the origin $O^{\prime }$. For convenience, we convert the image points in *O* and $O^{\prime }$ into unit vectors as

(4) $$m=\frac{1}{\sqrt{x^{2}+y^{2}+f^{2}}}\left(\begin{array}{c} x\\ y\\ f \end{array}\right)$$

(5) $$m^{\prime }=\frac{1}{\sqrt{x^{\prime 2}+y^{\prime 2}+f^{2}}}\left(\begin{array}{c} x^{\prime }\\ y^{\prime }\\ f \end{array}\right).$$

Thus, Equation (3) can be rewritten as

(6) $$r^{\prime }m^{\prime }=R^{-1}\left(rm-h\right)$$

where *r* and $r^{\prime }$ are the distances between the world point *P* and the camera at location *O* and $O^{\prime }$, respectively. As proven in [7], solving Equation (6) using the least square method, we can find the optimal solution for *r* and $r^{\prime }$ that minimizes the error between the two sides of the equation. The final solution is obtained as

(7) $$r=\frac{\left(h,m\right)-\left(m,{\mathrm{Rm}}^{\prime }\right)\left(h,{\mathrm{Rm}}^{\prime }\right)}{1-{\left(m,{\mathrm{Rm}}^{\prime }\right)}^{2}}$$

(8) $$r^{\prime }=\frac{\left(m,{\mathrm{Rm}}^{\prime }\right)\left(h,m\right)-\left(h,{\mathrm{Rm}}^{\prime }\right)}{1-{\left(m,{\mathrm{Rm}}^{\prime }\right)}^{2}}$$

where (·,·) is the inner product of two vectors.

With Equations (7) and (8), the same feature point appears in the previous frame and the current frame can be reconstructed. The remaining issues pertain to how the translation vector h, rotation matrix R, and unit vectors m and $m^{\prime }$ can be retrieved.

## 3. Visual Odometry

Visual odometry (VO) refers to computing the translation and rotation of a camera mounted on a vehicle by analyzing the associated camera images. Conventional methods such as the 8-point algorithm [8], the 7-point algorithm [9], and the 5-point algorithm [10] are all based on image feature detection and matching to compute the essential matrix, from which the rotation and translation (up to scale) are then recovered. Although these methods are now widely used with UGVs and UAVs, many strong features need to be detected and matched to render a good estimation of VO. However, in the case of sea environments, there are usually very few features that can be detected, because the sea water and sky take up most of the image, and their appearance are mostly uniform. Though some features can be detected on the wave, they are not stable and produce unreliable feature matches. For features detected on the sky (clouds), though they can be taken as stable points (since the clouds move very slow and can be considered as stationary in a short time period), they are too far away and cannot easily recover the translation of the camera accurately. Therefore, conventional VO methods are not suitable for USVs in the sea environment.

Using measurement sensors of the GPS and the IMU, we can directly obtain the motion of the camera. However, in our experiments, we use simple and cheap measurement sensors, the accuracy of which is not that high. To solve this problem, we propose using the horizon line to recover the roll and pitch angles in the rotation. The yaw angle is measured by the magnetometer sensor in our IMU, which works as a compass. The translation of the USV is computed directly from the recorded GPS data. To reduce the error produced by GPS, we take two frames with a large baseline (translation) to reconstruct the feature points, so that the influence of GPS error can be weakened.

**Figure 3:**
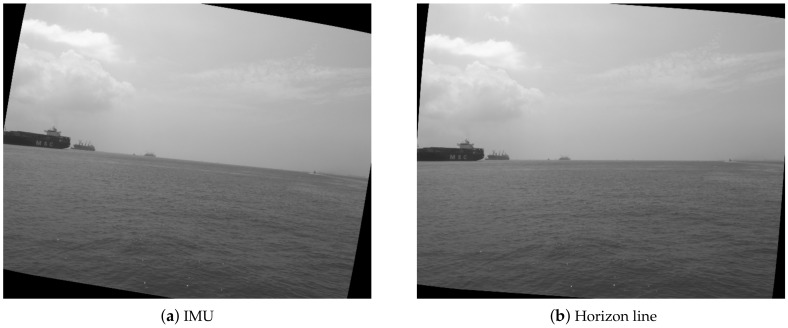
Roll correction using the IMU and the horizon line.

In the IMU (myAHRS+) used in our system, the yaw angle is measured by a magnetometer sensor, which is more reliable. While the roll and pitch angles are measured by a gyroscope and accelerometer, which are less reliable through our comparison in experiments. Figure 3a shows the roll angle corrected image using the measurement from the IMU. It can be seen that the horizon line in the image is not horizontalized well.

To compensate the low accuracy of roll and pitch angles rendered by the IMU in our system, we propose computing the roll and pitch from the horizon line. Therefore, the first step is to accurately detect the horizon in the image. In this work, structured edge detection [11] is applied to derive the edge map of the original image. After that, the RANSAC [12] method is used to fit a straight line (horizon) from the edge points.

**Figure 4:**
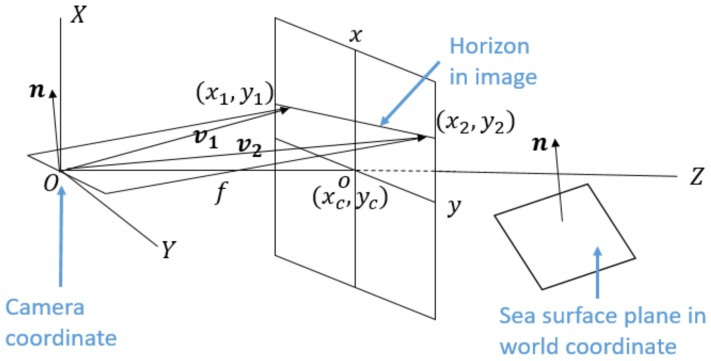
Sea surface plane estimation from the horizon line.

As shown in Figure 4, with the horizon or vanishing line, the normal vector n of the sea surface plane in the camera coordinate can be calculated, and it is the same as the normal vector in the world coordinate. Denote the image center as $\left(x_{c},y_{c}\right)$, the focal length of the camera as *f*, and two different points on the horizon line as $\left(x_{1},y_{1}\right)$ and $\left(x_{2},y_{2}\right)$. Thus, the two vectors connecting the origin of the camera coordinate and the two different points on the horizon line are, respectively, $v_{1}={\left(x_{1}-x_{c},y_{1}-y_{c},f\right)}^{T}$ and $v_{2}=(x_{2}-x_{c},$ $y_{2}-y_{c}{,f)}^{T}$. Hence, the normal vector of the sea surface plane is calculated as the cross product of $v_{1}$ and $v_{2}$: $n=v_{1}\times v_{2}={\left(n_{1},n_{2},n_{3}\right)}^{T}$. Finally, the roll α and pitch β angles of the camera can be computed as

(9) $$\alpha =\arctan\left(\frac{n_{1}}{n_{2}}\right),$$

(10) $$\beta =\arctan\left(\frac{n_{3}}{\sqrt{n_{1}^{2}+n_{2}^{2}}}\right).$$

**Figure 5:**
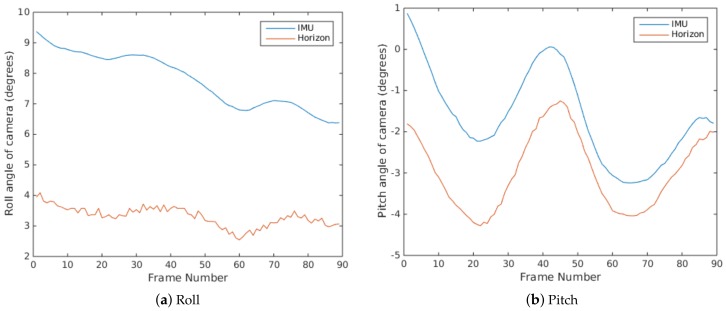
Roll and pitch angles of camera obtained from the IMU and horizon line.

Figure 5 illustrates the comparisons of roll and pitch angles obtained from the IMU and the horizon line. One can see that there are large gaps between the rotation measurements from the IMU and the horizon line. Furthermore, It can be seen in Figure 3, the image after roll correction using the horizon line are much better than that using the IMU. Therefore, in this work, we chose to derive the roll and pitch angles of the camera mounted on the USV from the horizon line instead of the IMU.

## 4. From 3D to 2D

The roll and pitch angles of the camera were obtained. Afterwards, the angle that was capable of correcting the image so that both the roll and pitch angles were equal to 0 was determined. This meant that the USV was moving on a 2D flat plane. Thereby, our problem can be simplified to the 2D case by roll and pitch correction. On the 2D plane, there exists only the yaw rotation between two frames.

Suppose that the rotation matrices for roll and pitch correction in the previous and current states are $R_{1}$ and $R_{1}^{\prime }$, respectively, and the relative rotation matrix of yaw angle from previous state to current state is $R_{2}$. Thus, Equations (7) and (8) can be rewritten as

(11) $$r=\frac{\left(h,R_{1}m\right)-\left(R_{1}m,R_{2}R_{1}^{\prime }m^{\prime }\right)\left(h,R_{2}R_{1}^{\prime }m^{\prime }\right)}{1-{\left(R_{1}m,R_{2}R_{1}^{\prime }m^{\prime }\right)}^{2}}$$

(12) $$r^{\prime }=\frac{\left(R_{1}m,R_{2}R_{1}^{\prime }m^{\prime }\right)\left(h,R_{1}m\right)-\left(h,R_{2}R_{1}^{\prime }m^{\prime }\right)}{1-{\left(R_{1}m,R_{2}R_{1}^{\prime }m^{\prime }\right)}^{2}}.$$

In Equations (11) and (12), h is the translation vector in the camera coordinate; however, we can only obtain the translation vector H in the world coordinate from the GPS. If we consider the 2D case and suppose $h={\left(X_{c},Z_{c}\right)}^{T}$, $H={\left(X_{w},Z_{w}\right)}^{T}$, then, knowing the yaw angle θ of the camera to the world north from the compass, h can be computed as

(13) $$\begin{array}{c} h=\left[\begin{array}{c} X_{c}\\ Z_{c} \end{array}\right]=\left[\begin{array}{c} \cos(\theta )\;\;\;\;\sin(\theta )\\ -\sin(\theta )\;\;\cos(\theta ) \end{array}\right]\left[\begin{array}{c} X_{w}\\ Z_{w} \end{array}\right]. \end{array}$$

## 5. Feature Matching

The matched feature points in a pair of images are generally found in three steps: feature detection, feature tracking, and feature matching.

**Feature detection.** To detect features from the image, we use ORB (oriented FAST and rotated BRIEF) [13], which is basically a fusion of the FAST keypoint detector [14] and the BRIEF descriptor [15], with many modifications to enhance the performance. It first uses FAST to find keypoints and then applies the Harris corner [16] measure to find the top *N* points among them. It also uses pyramids to produce multi-scale features. Its descriptor is the modified BRIEF descriptor.

**Feature tracking.** The detected features are tracked by calculating their optical flow using the iterative Lucas–Kanade method with pyramids [17].

**Feature matching.** To prevent feature drift during tracking, we perform feature matching for the tracked features in the pair of images that are selected to reconstruct the feature points. In this way, the drifted features can be filtered out, and only features with strong matching (closer distance) are retained. The matching process is performed in a brute-force manner.

**Figure 6:**
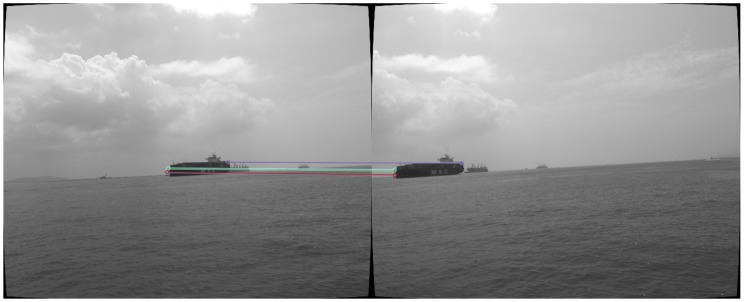
ORB feature detection, tracking, and matching. The straight lines in the image connecting the matched features.

Figure 6 shows the final matched features in a pair of images. It can be observed that the features are mainly located at the strong corners of the obstacle.

## 6. Obstacle Mapping

Points reconstructed from the current frame with only one pair of frames may not be reliable or accurate enough, due to the errors from different sources, including feature matching, horizon detection, GPS, and IMU sensors. To improve the accuracy of reconstruction, we propose reconstructing points in the current frame with multiple pairs of frames.

Denote the current frame as $I_{c}$, and several previous frames as $I_{i},i=\left(1,2,\ldots ,m\right)$, where *m* is the total number of selected previous frames. With each pair of frames $\left\{I_{c},I_{i}\right\}$, the depth $r_{ci}$ and $r_{ci}^{\prime }$ of a pair of matched feature points in the previous and current frames can be triangulated using Equations (11) and (12), respectively. Knowing the locations in the world coordinate of the pair of frames from GPS, together with $r_{ci}$ and $r_{ci}^{\prime }$, the 3D location $P_{ci}$ of this matched feature point in the world coordinate can be calculated by basic techniques of triangle geometry.

Therefore, in total we can obtain *m* measurements for the same feature point in the current frame. Then, we give each of the *m* measurements a weight $\omega_{ci}$, and the final determined 3D location $P_{c}$ of this feature point is synthesized by computing the weighted sum of the *m* measurements. This process is formulated by the following equation:

(14) $$\left\{\begin{array}{c} P_{c}=\sum_{i=1}^{m}\omega_{ci}P_{ci},\\ \sum_{i=1}^{m}\omega_{ci}=1. \end{array}\right.$$

Finally, the obstacle map is built by projecting the reconstructed 3D points using Equation (14) to a grid map or the commercial product, Google Maps.

## 7. Experimental Results

The proposed wide-baseline stereo system in this work is composed of a Point Grey grasshopper3 CCD camera with an image size of 2736×2192, a frame rate of 13 Hz, and a focal length of 2840, a camera lens with a horizontal field of view (FOV) of 50 degrees, a Trimble SPS351 GPS unit with a frequency of 10 Hz, a myAHRS+ sensor module with a frequency of 100 Hz, and a laptop equipped with i7, 2.60 GHz CPU.

Since the frequency of GPS is lower than that of the camera, we do a linear interpolation as formulated in Equation (15) so that each frame has GPS data.

(15) $$L_{img}=\frac{T_{img}-T_{GPS1}}{T_{GPS2}-T_{GPS1}}\left(L_{GPS2}-L_{GPS1}\right)+L_{GPS1}$$

where $L_{img}$ is the interpolated location of the current frame, $T_{img}$ is the time stamp of current frame, $T_{GPS1}$ and $T_{GPS2}$ are the GPS time stamp that are before and after $T_{img}$, respectively, and $L_{GPS1}$ and $L_{GPS2}$ are the respective GPS locations at $T_{GPS1}$ and $T_{GPS2}$.

The myAHRS+ sensor has a high frequency, so we simply find the yaw data that has closest time stamp to that of the video frame and assign it to that video frame.

### 7.1. Dataset

Since there is no public dataset for vision-based obstacle mapping in a maritime environment, we evaluate the proposed algorithm on our own dataset, which includes three image sequences (Seq-1, Seq-2, and Seq-3) captured using the proposed wide-baseline stereo system from a moving USV in open sea. The dataset is characterized as follows:

- Seq-1 contains 100 continuous gray frames. A stationary buoy about 100 m away from the USV appears in each frame.
- Seq-2 contains 90 continuous gray frames. A stationary tanker about 500 m away from the USV is shown in each frame.
- Seq-3 is the full sequence, from which Seq-1 and Seq-2 are cut out. This sequence contains 1185 continuous gray frames, and presents the scenes while the USV is travelling along a circular route on the sea.

Seq-1 and Seq-2 are used for quantitative evaluation of the stability of our proposed reconstructing approach that leverages multiple frame pairs. Seq-3 is used for visual evaluation of the performance of the proposed obstacle mapping with a full moving loop of USV.

We do not currently have ground truth locations of the obstacles in this dataset, so we now evaluate the stability of our proposed approach.

### 7.2. Performance Evaluation

Here, we evaluate the stability of our proposed system by examining the variance of the mapped feature points in our dataset. In the following experiments, we take equal weights in Equation (14) for a simple evaluation.

First, we need to find a good value of *m* in Equation (14), so that reliable reconstruction results can be obtained. Table 1 shows our trials with Seq-1. We select two feature points from the obstacle in the image, and each of these two features are tracked frame by frame and reconstructed to the geodetic coordinate (latitude and longitude) with different numbers of frame pairs. Finally, the variances of the reconstructed locations with each feature in the latitude (Var Lat) and longitude (Var Lon) dimensions are calculated, respectively. One can deduce from Table 1 that the reconstruction with m=5 and m=10 pairs of frames lead to smaller variances than that with m=1 or m=15. The reason is that the reconstruction using only one pair of frames is not reliable enough, while using too many pairs of frames may degrade the accuracy of the final reconstruction result. For instance, frame pairs with a short baseline usually lead to large errors when distant points are reconstructed.

**Figure 7:**
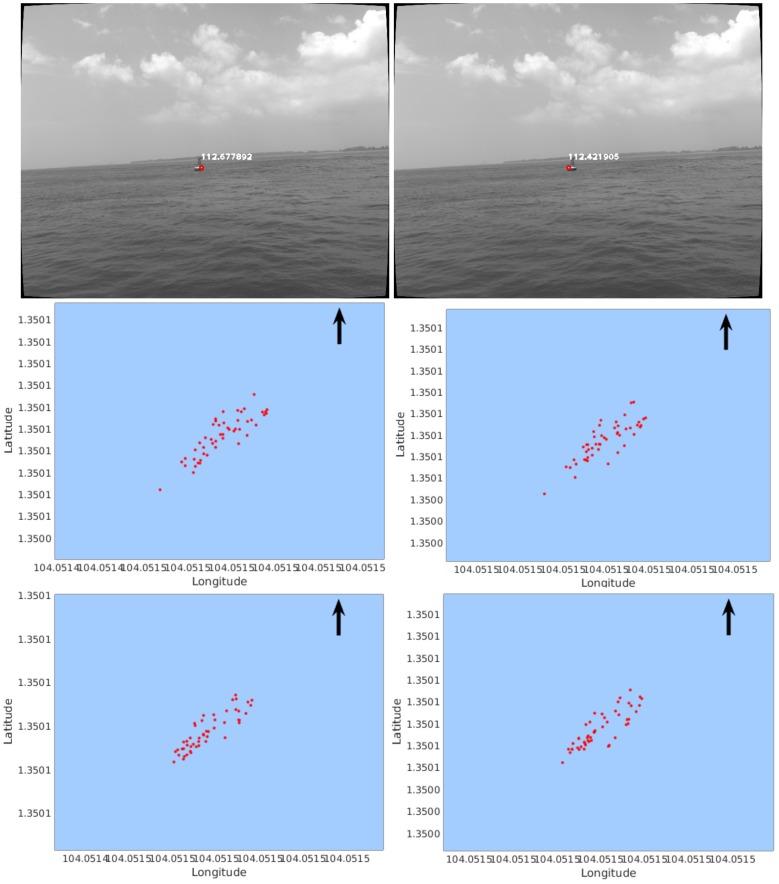
Distribution of mapped feature points (red) in Seq-1. (The arrow points to the direction of North.) The left column shows the case of Feature Point #1; the right column shows the case of Feature Point #2. The top row shows the features on images with their distances displayed in meters. The middle row shows the reconstructed points using one pair of frames. The bottom row shows the reconstructed points using five pairs of frames.

The top row of Figure 7 illustrates the two features mentioned in Table 1. As can be seen, the two features are extracted from different parts of the buoy, and their estimated distances are displayed. Comparing the distributions of mapped feature points in the middle row to that in the bottom row, we can observe that the feature maps reconstructed from five pairs of frames are less scattered than that with one frame pair.

**Figure 8:**
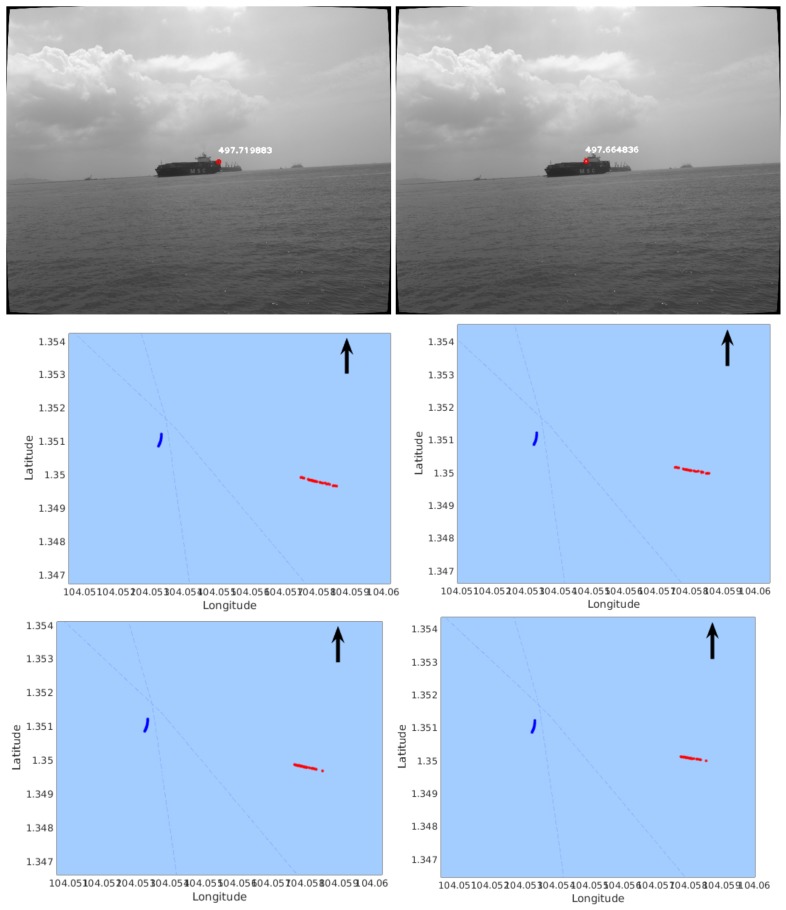
Distribution of mapped feature points (red) in Seq-2. (The blue curve represents the trajectory of our USV; The arrow points to the direction of North.) The left column shows the case of Feature Point #1; the right column shows the case of Feature Point #2. The top row shows the features on images with their distances displayed in meters. The middle row shows the reconstructed points using one pair of frames. The bottom row shows the reconstructed points using ten pairs of frames.

Similar to the experiments in Seq-1, we also tried different *m* values with Seq-2. It can be seen in Table 2 that smaller location variances can be obtained by using ten pairs of frames for reconstruction. Furthermore, Figure 8 shows the two features in Table 2 and compares the distributions of mapped points using one frame pair (middle row) with that using 10 frame pairs (bottom row), which again demonstrates less scattered results.

**Figure 9:**
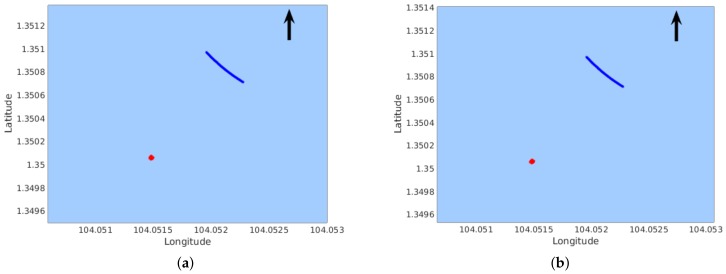
Obstacle mapping result of Seq-1. The red points are the reconstructed feature points from obstacles; The blue curve represents the trajectory of our USV; The arrow points to the direction of North. (**a**) Triangulation using one frame pair; (**b**) triangulation using ten frame pairs.

**Figure 10:**
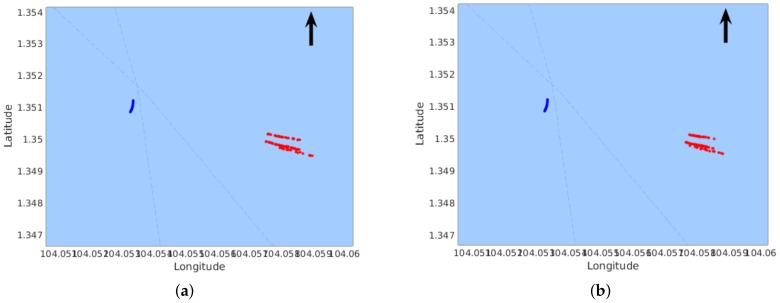
Obstacle mapping result of Seq-2. The red points are the reconstructed feature points from obstacles; The blue curve represents the trajectory of our USV; The arrow points to the direction of North. (**a**) Triangulation using one frame pair; (**b**) triangulation using ten frame pairs.

Figure 9 and Figure 10 show the final results of obstacle mapping with Seq-1 and Seq-2, respectively. Our proposed approach of leveraging multiple frame pairs to reconstruct feature points can be compared with that using a single frame pair. The difference is hard to detect in Figure 9, because the approach is more reliable with near-field obstacles, which have a larger position difference in the frame pairs and thus more tolerant to noise. Thus, the difference between using a single frame pair and using multiple frame pairs is very small. However, in Table 1, this difference can be seen. The obstacle in Seq-2 is a distant tanker, so it can be observed in Figure 10 that the distribution of reconstructed features points using multiple frame pairs is less scattered than that using a single frame pair.

**Figure 11:**
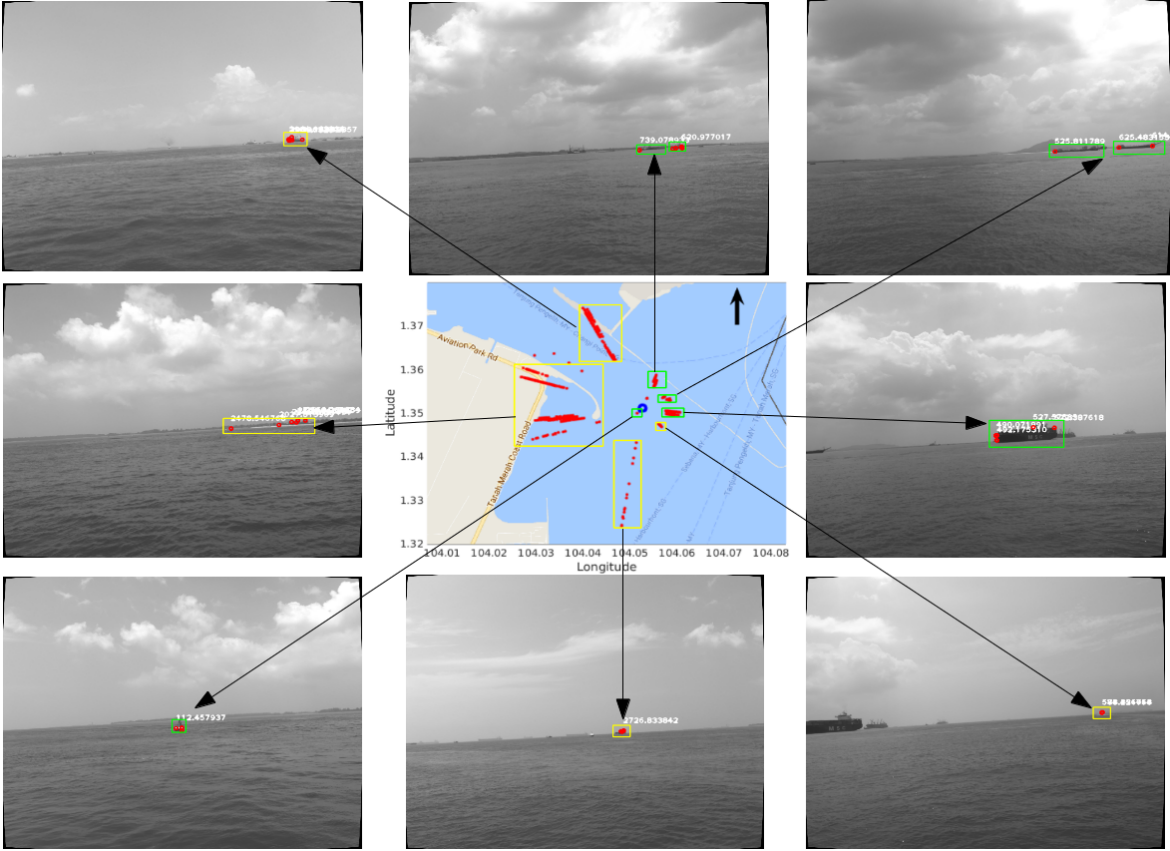
The resulting obstacle map (middle) after a full moving loop of the USV (Seq-3). The corresponding obstacles in the original images are shown in the surrounding figures with an arrow linking each of them to the map. In the obstacle map, the blue circle represents the trajectory of the USV, and the red points represent the mapped feature points from the obstacles. The green rectangles are manually drawn to illustrate the stationary obstacles, while the yellow rectangles are manually drawn to show the distant obstacles with large mapping variances and the moving obstacles.

The resulting obstacle map with Seq-3 is shown in the middle figure of Figure 11, and the corresponding obstacles in the original images are shown in the surrounding figures. This obstacle map is obtained with m=10 and a range limit of 3000 m. It can be seen that some of the stationary obstacles can be effectively mapped after a full travelling loop of the USV. In Figure 11, the bottom-left image shows a stationary buoy with a measured distance of around 100 m, and its corresponding points in the obstacle map have a very small variance, due to the fact that, as distance decreases, the strength of the detected features become stronger, and the feature matching becomes more accurate. The images in the middle-right, top-right, and top-middle show stationary tankers and boats with a distance to the USV of around 500 m, and these obstacles demonstrate larger but acceptable variances in the obstacle map. A large container ship is shown in both the bottom-middle and top-left images with very large distances (>1000 m) to the USV, and they are mapped with very large variances, because the detected features from the distant obstacles are very weak and cannot be easily matched correctly, resulting in unreliable distance estimation. A similar case also appears in the middle-left image, which shows the scenario of the seashore. A moving obstacle case is illustrated in the bottom-right image, which contains a fast moving boat with a computed distance of around 500 m; however, by visually comparing this boat with the stationary tanker (an estimated distance of around 500 m) on the left of this image, one can observe that the moving boat is much farther away from the USV than the tanker, so the mapped points from this moving boat are not reliable.

Therefore, it can be concluded from Figure 11 that the proposed obstacle mapping system for USVs is very sensitive to feature matching. Nearby stationary obstacles can be robustly mapped with small variances, because strong features can be detected and then matched with high accuracy, while distant stationary obstacles might be mapped with large variances due to weak features and less reliable matching. However, regardless of feature strength, moving obstacles will be incorrectly mapped. Another important reason for the large mapping variance of distant obstacles lies in the inherent nature of stereo vision. In Equation (2), $\Delta Z\propto Z^{2}$. Thus, a greater depth yields a greater reconstruction error, which results in the larger variance in obstacle mapping. The reliable mapping range of the proposed system is from 100 to 1000 m.

## 8. Conclusions

A new approach for wide-baseline stereo-based obstacle mapping for USVs has been presented. In the proposed approach, the VO of the camera is computed from a horizon line, a magnetometer, and GPS data. Via VO, the reconstruction of feature points are simplified to 2D space, and its reliability is enhanced using multiple frame pairs. Finally, the reconstructed feature points are projected to a Google Map.

The proposed approach gets rid of the bulky structure and difficult calibration of binocular stereo. On the contrary, it is very convenient to setup and requires only simple calibration for the intrinsic parameters of the camera. Its ranging ability is approximately 100–1000 m. However, it can only reconstruct points from static scenes, and the USV should be in a state of travel. Furthermore, measurement noise from the GPS, the compass, horizon detection, and especially feature matching might influence the accuracy of this approach.

The proposed approach requires the horizon to be detected. Future work could consist in detecting the water line that segments the sea surface from the seashore, akin to the work of [18], so that the proposed approach could work when the camera is nearby and facing the seashore. Another future work could involve integration with image-based methods for obstacle detection, so that the points in the resulted obstacle map can be easily clustered. To build a more robust system, it is also important to detect and map moving obstacles. More quantitative evaluation for the proposed method will be conducted in the future via comparison with logged radar data (ground truth).

## Figures and Tables

**Table 1:** A reconstructed location (latitude and longitude) variances of two feature points in Seq-1 using different numbers (*m*) of frame pairs. The smallest variance value in each column is highlighted with bold typeface.

*m*	Feature Point #1		Feature Point #2	
	Var Lat ($\times 10^{-10}$)	Var Lon ($\times 10^{-10}$)	Var Lat ($\times 10^{-10}$)	Var Lon ($\times 10^{-10}$)
1	0.2211	0.3366	0.1907	0.2953
5	**0.1764**	0.2758	**0.1679**	**0.2568**
10	0.2062	**0.2723**	0.2232	0.2573
15	0.2483	0.2733	0.2939	0.2741

**Table 2:** Reconstructed location (latitude and longitude) variances of two feature points in Seq-2 using different numbers (*m*) of frame pairs. The smallest variance value in each column is highlighted with bold typeface.

*m*	Feature Point #1		Feature Point #2	
	Var Lat ($\times 10^{-7}$)	Var Lon ($\times 10^{-7}$)	Var Lat ($\times 10^{-7}$)	Var Lon ($\times 10^{-7}$)
1	0.0555	0.9332	0.0288	0.8539
5	0.0291	0.5546	0.0140	0.4926
10	**0.0194**	**0.4209**	**0.0095**	**0.3895**
15	0.0441	0.7795	0.0226	0.7079
